# Comparison of Hemostatic Efficacy of Argon Plasma Coagulation with and without Distilled Water Injection in Treating High-Risk Bleeding Ulcers

**DOI:** 10.1155/2014/413095

**Published:** 2014-08-27

**Authors:** Yuan-Rung Li, Ping-I Hsu, Huay-Min Wang, Hoi-Hung Chan, Kai-Ming Wang, Wei-Lun Tsai, Hsien-Chung Yu, Feng-Woei Tsay

**Affiliations:** ^1^Division of Gastroenterology, Department of Internal Medicine, Kaohsiung Veterans General Hospital and National Yang-Ming University, Kaohsiung 81362, Taiwan; ^2^Department of Biological Sciences, National Sun Yat-Sen University, Kaohsiung 80424, Taiwan

## Abstract

*Background*. Argon plasma coagulation (APC) is useful to treat upper gastrointestinal bleeding, but its hemostatic efficacy has received little attention. *Aims*. This investigation attempted to determine whether additional endoscopic injection before APC could improve hemostatic efficacy in treating high-risk bleeding ulcers. *Methods*. From January 2007 to April 2011, adult patients with high-risk bleeding ulcers were included. This investigation compared APC plus distilled water injection (combined group) to APC alone for treating high-risk bleeding ulcers. Outcomes were assessed based on initial hemostasis, surgery, blood transfusion, hospital stay, rebleeding, and mortality at 30 days posttreatment. *Results*. Totally 120 selected patients were analyzed. Initial hemostasis was accomplished in 59 patients treated with combined therapy and 57 patients treated with APC alone. No significant differences were noted between these groups in recurred bleeding, emergency surgery, 30-day mortality, hospital stay, or transfusion requirements. Comparing the combined end point of mortality plus the failure of initial hemostasis, rebleeding, and the need for surgery revealed an advantage for the combined group (*P* = 0.040). *Conclusions*. Endoscopic therapy with APC plus distilled water injection was no more effective than APC alone in treating high-risk bleeding ulcers, whereas combined therapy was potentially superior for patients with poor overall outcomes.

## 1. Introduction

Acute upper gastrointestinal bleeding (UGIB) is common and has significant associated mortality and morbidity. Up to 5–10% mortality of upper gastrointestinal bleeding results from bleeding event or worsening of concurrent medical illness [1, 2]. Severe upper gastrointestinal bleeding typically results from peptic ulcer bleeding, and endoscopic treatment can effectively reduce the rate of rebleeding, the need for surgery, and the mortality [3].

Effective endoscopic therapies for bleeding ulcers include injection with sclerosants/epinephrine/normal saline, contact thermal coagulation, and hemoclips [4]. Endoscopic injection therapy is recommended by studies owing to the tamponade effect in hemostasis [5]. Injection therapy is clearly effective, easy to administer, and relatively inexpensive. Additionally, injection therapy can slow bleeding and facilitate other treatments. Thermal coagulation uses heat probe or argon plasma coagulation (APC) device for hemostasis. Heat probe coagulation uses a device that directly contacts the point where bleeding is occurring to achieve thrombosis and coagulation. Meanwhile, APC is a noncontact method that uses high-frequency monopolar current associated with ionized and electrically conductive argon gas [6].

Combination therapy outperforms single therapy for hemostasis. For example, hemoclips combined with injection therapy outperform either hemoclips or injection therapy alone [7, 8]. Injection therapy combined with heat probe treatment outperforms injection therapy alone but does not differ significantly from heater probe therapy alone [9]. This is probably because heat probe coagulation exerts a tamponade effect via direct contact [9].

Theoretically, no tamponade effect exists for APC alone. This study aimed to determine whether additional endoscopic injection before APC could increase hemostatic efficacy in treating high-risk bleeding ulcers.

## 2. Methodology

### 2.1. Patient

From January 2007 to April 2011, a retrospective analysis was performed of patients hospitalized due to upper gastrointestinal bleeding. Inclusion criteria were as follows: (1) patients with melena or hematemesis and (2) patients in which the emergent upper endoscopy revealed high-risk bleeding ulcers within 24 hours upon admission to the emergency units. Exclusion criteria included (1) another possible bleeding site, (2) coexistence of severe illness (e.g., acute stroke, acute surgical abdomen, acute myocardial infarction, or sepsis), (3) pregnancy, (4) patient age under 20 years old, and (5) tendency to systemic bleeding (e.g., prothrombin time > 3 sec, platelet count < 50,000/mm^3^, or treatment with an anticoagulant agent).

High-risk bleeding ulcers were defined as those with stigmata of an actively bleeding visible vessel (i.e., spurting or oozing), a nonbleeding visible vessel (NBVV), or adherent clots [10]. A NBVV was defined as a raised red or bluish-red hemispheric vessel protruding from the ulcer base, without active bleeding. Meanwhile, an adherent clot was defined as an overlying clot that was wash-resistant.

### 2.2. Endoscopic Treatment

All patients were admitted to our emergency department with nonvarices upper gastrointestinal bleeding. They received an intravenous bolus of 40 mg pantoprazole, followed by panendoscopy within 24 hours upon admission. Local pharyngeal anesthesia was used by 8% xylocaine spray, gastric lavage before endoscopy to enhance the visual field, and intramuscular injection with hyoscine methonitrate 20 mg for premedication. Patients with high risk of bleeding ulcers nonrandomly received either APC plus distilled water injection (combined group) or APC alone (APC group).

Therapeutic endoscopies were performed by four experienced endoscopists with more than 3 years of experience, using Olympus GIF XV10, GIF XQ200, and GIF 1T20 (Olympus Corporation, Tokyo, Japan) devices. Stigmata of active bleeding ulcer or adherent blood clots were irrigated with distilled water via the accessory channel of the endoscopy [11]. A large blood clot that covered the ulcerative lesion was removed using a 3-prong device, snare catheter, or water irrigation. Distilled water was then injected in aliquots of 0.5 to 2.0 mL, at and around the suspected site of bleeding, up to 20.0 mL if needed. The injections were placed in the 4 quadrants surrounding the bleeding site or the vessel. No bleeding was noted after injection for at least 3 minutes. Injection amount was determined by endoscopists. APC was performed by an Olympus electrosurgical unit/APC unit (PSD-60/Endoplasma, Olympus Corp., Tokyo, Japan), and its catheters were 2.3 mm and 3.5 mm and were equipped with endoscope channels with corresponding diameters [11]. APC used a coagulation mode at a gas flow/power setting of 1.5 L/min and 40~60 watts (40 watt for duodenal ulcer; 40~60 watt for gastric ulcer). Operative distance between the probe and suspected site of bleeding ranged from 2 to 8 mm.* Helicobacter pylori* status was not verified during acute bleeding episodes since, according to several studies, the biopsy-based test must have a low sensitivity to detect* H. pylori* in bleeding ulcers [12].

Initial hemostasis was defined as following the first endoscopic treatment (index endoscopy) and endoscopically verified to have stopped bleeding for at least 5 minutes. If the initial hemostasis failed owing to uncontrollable profusion, patients received subsequent endoscopic modality or emergency surgery, as determined by a gastroenterologist. Recurrent hemorrhaging during a 30-day observation period, defined herein as rebleeding, includes one or more of the following factors: aspiration of fresh blood from a nasogastric tube, new hemostasis event, pulse rate over 100 beats/min with unstable vital signs, drop in systolic blood pressure exceeding 30 mmHg, or continuous melena with drop in Hb of at least 2 g/dL. Upper endoscopy was performed immediately if rebleeding occurred, followed by second hemostasis. Both treated groups with recurrent bleeding underwent endoscopic combination therapy, either APC plus distilled water injection or hemoclipping plus distilled water injection as a rescue therapy. If the second endoscopic therapy failed to achieve hemostasis, emergency surgery was performed.

### 2.3. Medication Treatment and Follow-Up

Medical treatments included partial parenteral nutrition and intravenous administration of pantoprazole (40 mg every 24 h) and the patients continued to fast [13, 14]. Following two days of observation, the patients consumed a soft diet for 2 to 3 days, followed by a regular diet; pantoprazole was shifted to an oral form (40 mg daily). For the first three days, daily hemoglobin (Hb) levels were monitored routinely for following the index endoscopy. Blood transfusion criteria included the following: (1) persistent hematemesis or melena, with a systolic blood pressure below 100 mm Hg or a pulse rate exceeding 100 beats/min, and (2) Hb levels lower than 8 g/dL.

Patients received oral pantoprazole (40 mg daily) for up to 8 weeks following discharge and were instructed to undergo follow-up through our outpatient department on days 14, 28, and 56 after the initial hemostasis was achieved.

### 2.4. Analysis

Qualitative variables, similar to the baseline characteristics and treatment outcomes, were compared via the *Χ* ^2^ and Fisher exact tests. Quantitative variables were also compared using Student's *t*-test, and the data were expressed as mean ± SD. Next, the risk factors for the development of rebleeding on univariate and/or multivariate analysis were evaluated using the Cox regression model. Those variables significant at *P* < 0.20 in the univariate models were subsequently subjected to multivariate analysis in order to identify the most significant predictors. All hypothesis tests were compared with a two-sided alternative, whenever deemed appropriate. The level of statistical significance was set at *P* < 0.05. Analyses were performed using SPSS software (SAS, SPSS Inc., Chicago, IL).

## 3. Results

One hundred thirty-five patients were included in the study between January 2007 and April 2011 and were recruited through the Division of Gastroenterology, Department of Internal Medicine, Kaohsiung Veterans General Hospital. Fifteen patients were excluded owing to gastric cancer (*n* = 2), acute severe illness (*n* = 10), and bleeding tendency (*n* = 3). Finally, 60 patients underwent combination therapy (APC plus distilled water injection) and 60 patients received APC alone.

Among the 120 patients with ulcers who had a high risk of bleeding, most of them are male (79.1%) or over 60 years old (69.1%). The sample included 70 cases of bleeding duodenal ulcer (34 who received combined therapy and 36 who received APC alone) and 41 cases of bleeding gastric ulcer (23 who received combined therapy and 18 who received APC alone) and nine cases of bleeding stump ulcer (three who received combined therapy and six who received APC alone) were included.  Table 1 lists the clinical data of the patient on study entry. The two treatment groups were similar in terms of all baseline characteristics.

Initial hemostasis was achieved in 98.3% (59/60) and 95.0% (57/60) of the combined group and APC alone group, respectively, with similar initial hemostasis rates (*P* = 0.619). Bleeding recurred in two patients (3.4%) treated with combined therapy and in seven (12.3%) treated with APC alone. Subsequently, one patient underwent APC plus distilled water injection and the other received hemoclipping plus distilled water injection in the combined group. In the APC group, two patients underwent APC plus distilled water injection and five patients received hemoclipping plus distilled water injection. Despite a higher rebleeding rate in the APC alone group, the two groups did not differ significantly (*P* = 0.092).  Table 2 lists the data on clinical outcomes for the patients in this study. Next, possible reasons for recurrent bleeding were examined. According to those results, NSAID use (*P* = 0.046) and previous bleeding (*P* = 0.045) may predict the possibility of rebleeding, based on Cox regression multivariate analysis (Table 3). One patient (1.7%) in the combined group and five (8.3%) in the APC group underwent emergency surgery (*P* = 0.207). The two treatment groups exhibited no significant differences in 30-day mortality (1.7% versus 3.3%, *P* = 1.000), hospital stay (6.6 + 4.5 versus 5.7 + 2.9 days, *P* = 0.219), or transfusion requirements (4.3 + 4.2 versus 3.6 + 2.7 units, *P* = 0.306). Moreover, both groups were free of major complications. The combined treatment group only exhibited an advantage (5.0% versus 16.7%, *P* = 0.040) in the combined end point of mortality, as well as the failure of initial hemostasis, rebleeding, and the need for surgery.

Two of the three deaths occurred in patients with uncontrollable bleeding, with both of those patients belonging to the APC group. The other one occurred in the patient with progressive pneumonia and septic shock. No life-threatening procedure-related complications were observed in either group at index endoscopy. However, three patients with NBVV (3/23, 13%) in the APC group and none (0/25, 0%) in the combined treatment group experienced procedure-induced bleeding. Fortunately, these adverse events were subsequently controlled through repeated APC therapy.

## 4. Discussion

Thus far, despite the effectiveness of endoscopic injection therapies, the rebleeding rate remained around 20% [22]. Recurrent bleeding has been reported to be the most important factor in predicting mortality [23]. Thus, several endoscopic methods for hemostasis of gastrointestinal bleeding have been developed, including heat probe coagulation, mechanical devices like hemoclips, and APC.

Injection therapy is frequently used as an endoscopic treatment for ulcer bleeding, with 1 : 10000 diluted epinephrine as the injected solution. Because mechanical compression of injected solution is the most significant factor for initial bleeding control, some studies have demonstrated that large volume endoscopic injection therapy can help prevent rebleeding via the same mechanism as compression effect [24, 25]. Regarding the reason for using distilled water rather than epinephrine injection, some scholars agreed that epinephrine injection may result in increased risk of cardiovascular event. Sung and his colleagues [26] found that plasma epinephrine concentration increased significantly for 10 minutes following endoscopic injection and increased the likelihood of adverse cardiovascular events. Distilled water is used rather than epinephrine injection because epinephrine injection may increase the risk of a cardiovascular event. Meanwhile, Lai et al. [5] found injection therapy with distilled water or 1 : 10000 diluted epinephrine did not significantly differ from each other upon initial hemostasis. Endoscopic therapy with distilled water injection has traditionally been the most popular approach and continues to be considered safe and effective. Severe complications (e.g., perforation, worsening of bleeding) have not been reported in association with distilled water injection [5, 27]. Due to the above reason, we choose endoscopic injection with distilled water for tamponade effect.

Combination therapy is accepted to be better than single therapy in hemostasis. For example, the combination of injection therapy with another method of hemostasis (e.g., hemoclips or heat probe) outperforms injection alone for controlling bleeding, particularly high-risk bleeding [7, 8]. However, therapeutic gain of contact thermal therapy and injection therapies may display no more hemostatic benefits than contact thermal monotherapy does [9]. Because contact thermal therapy, such as heat probe, exerts a tamponade effect on the artery, coaptivity coagulates the tissue, activates arterial coagulation, and causes edema that compresses the artery, and it has similar hemostatic efficacy to combined therapy [28, 29].

APC, a noncontact thermal therapy, can more easily target the sites of bleeding than can hemoclips, particularly those in the posterior wall of lesser curvature of the upper gastric body or the posterior wall of the duodenal bulb [30, 31]. In spite of its efficient treatment of radiation proctitis, angiodysplasia, and gastric antral vascular ectasia, some trials have been published on the efficacy of APC for treating bleeding peptic ulcer. APC had similar efficacy to heat probe in terms of initial hemostasis and the prevention of recurrent bleeding [32, 33]. APC also achieved a lower rebleeding rate than distilled water injection therapy [11]. Moreover, APC had the same effects as contact thermal therapy, including tissue coagulation, arterial coagulation, and tissue edema, except the direct compression effect. The risk of perforation following APC is estimated at approximately 0.3% [33]. Although this potential risk can be considered a disadvantage of APC, both groups in the present were free of perforation, probably because of the noncontact method.

This investigation was specifically designed to test the hypothesis that outcomes differ between patients treated by APC monotherapy and combined APC therapy with distilled water injection. Combined therapy was expected to increase the local tamponade effect since APC lacks a direct compression effect. The results showed that both treatment arms were equally effective in terms of initial hemostasis (98.3% versus 95.0%). Notably, a trend existed toward lower recurrent bleeding (3.4% versus 12.3%, *P* = 0.092) in the combined group yet does not reach statistical significance. The tamponade effect with noncontact thermal therapy may affect recurrent bleeding rate. In the current study, the rate of peptic ulcer rebleeding was relatively low (3.4%) in the combined group, even though those patients did not receive high-dose PPI therapy. A lower incidence of Forrest grades IIa and IIb than other clinical trials may contribute to this phenomenon [7–9, 11]. No significant difference existed between the two treatment groups in terms of need for surgery, need for blood transfusion, hospital stay, and mortality. The combined group exhibited an advantage in the combined end point of mortality plus the failure of initial hemostasis, rebleeding, and the need for surgery (5.0% versus 16.7%, *P* = 0.040). In the future, larger randomized studies may be able to clarify the differences between these two treatment methods.

This investigation suffers some limitations. First, it includes possible selection bias. Volunteer participants for endoscopy may have a reason to suspect that they have other coexisting bleeding sites. Second, examiner subjectivity may influence endoscopic estimation of ulcer size. Third, this study excluded some patients unable to undergo endoscopy owing to acute critical illness or tendency of systemic bleeding. Finally, this study is not a randomized controlled study. However, all study participants were followed up using standard protocols administered by experienced gastroenterologists and trained assistants. Notably, this study was the first to investigate the effectiveness and safety of APC as an additional endoscopic treatment compared to APC alone in treating high-risk bleeding ulcers.

## 5. Conclusions

In conclusion, endoscopic therapy with APC plus distilled water injection failed to prove more effective than APC alone in treating high-risk bleeding ulcers, while APC plus injection therapy may be superior for patients with poor overall outcomes.

## Figures and Tables

**Table 1:** Baseline characteristics of the study group.

	Combined group (*n* = 60)	APC group (*n* = 60)	*P* value
Age, year (SD)	65.7 ± 17.2	68.0 ± 14.7	0.433
Age ≥ 60 yrs	40 (66.7%)	43 (71.7%)	0.553
Male gender	48 (80.0%)	47 (48.3%)	0.822
Cigarette consumption	13 (21.7%)	13 (21.7%)	1.000
Alcohol consumption	11 (18.3%)	7 (11.7%)	0.306
Aspirin use	3 (5.0%)	4 (6.7%)	1.000
NSAID use	27 (45.0%)	19 (31.7%)	0.113
Steroid use	1 (1.7%)	4 (6.7%)	0.364
Previous ulcer bleeding	19 (31.7%)	16 (26.7)	0.547
Hypovolemic shock	11 (18.3%)	14 (23.3%)	0.500
Hemoglobin, g/dL (SD)	10 (16.7%)	12 (20.0%)	0.637
Platelet count, k/cumm (SD)	200.5 ± 87.6	194.0 ± 63.8	0.644
Thrombocytopenia∗	16 (26.7%)	17 (28.3%)	0.838
PT/APTT prolongation	9 (15.0%)	6 (10.0%)	0.408
Comorbid disease∗∗	17 (28.3%)	18 (30.0%)	0.841
Ulcer size, mm (SD)	15.0 ± 7.1	16.0 ± 8.7	0.514
Ulcer ≥ 20 mm	18 (30.0%)	21 (35.0%)	0.559
Ulcer location			
Gastric ulcer	23 (38.3%)	18 (30.0%)	0.336
Duodenal ulcer	34 (56.7%)	36 (60.0%)	0.711
Stump ulcer	3 (5.0%)	6 (10.0%)	0.491
Bleeding type			
Spurting	3 (5.0%)	5 (8.3%)	0.717
Oozing	19 (31.7%)	26 (43.3%)	0.187
NBVV	25 (41.7%)	23 (38.3%)	0.709
Adherent clot	13 (21.7%)	6 (10.0%)	0.080
Injection volume, mL (SD)	8.4 ± 4.4		

APTT: activated partial thromboplastin time; NBVV: nonbleeding visible vessel; PT: prothrombin time; and SD: standard deviation.

∗Thrombocytopenia is defined as platelet count <150000/mm^3^.

∗∗Comorbid disease included old stroke, diabetes mellitus, liver cirrhosis, uremia, congestive heart failure, chronic pulmonary obstructive disease, and poststatus chemotherapy [15–21].

**Table 2:** Clinical outcomes of the study population.

	Combined group (*n* = 60)	APC group (*n* = 60)	*P* value
Initial hemostasis	59 (98.3%)	57 (95.0%)	0.619
30-day rebleeding	2 (3.4%)	7 (12.3%)	0.092
Rebleeding time			
Within 3 days	2 (3.4%)	6 (10.6%)
Between 4th and 30th days	0 (0%)	1 (1.8%)
Ulcer character		
Spurting	0 (0%)	1 (1.8%)
Oozing	1 (1.7%)	3 (5.3%)
NBVV	1 (1.7%)	2 (3.5%)
Adherent clot	0 (0%)	1 (1.8%)
Surgery	1 (1.7%)	5 (8.3%)	0.207
Blood transfusion, unit (SD)	4.3 ± 4.2	3.6 ± 2.7	0.306
Hospital stay, day (SD)	6.6 ± 4.5	5.7 ± 2.9	0.219
30-day mortality (SD)	1 (1.7%)	2 (3.3%)	1.000
Uncontrollable bleeding	0 (0%)	2 (3.3%)
Septic shock	1 (1.7%)	0 (0%)
Treatment failure	3 (5.0%)	10 (16.7%)	0.040

∗Treatment failure included initial treatment failure, rebleeding, surgery, and mortality.

**Table 3:** Probable effects of variables on recurrent bleeding.

	Univariate analysis		Multivariate analysis	
	OR (95% CI)	*P* value	OR (95% CI)	*P* value
Treatment method	0.30 (0.06–1.57)	0.155	0.09 (0.01–1.13)	0.062
NSAIDs use∗	5.03 (0.97–26.10)	0.055	8.90 (1.04–76.37)	0.046
Steroid use∗	4.95 (0.45–53.99)	0.189	7.05 (0.41–122.41)	0.180
Previous bleeding	4.48 (1.01–19.96)	0.049	7.50 (1.05–53.82)	0.045
Hypotension	4.35 (1.00–18.98)	0.050	0.86 (0.09–8.02)	0.897
Thrombocytopenia	10.38 (1.97–54.76)	0.006	6.32 (0.86–48.59)	0.071

CI: confidence interval; OR: odds ratio.

∗NSAIDs use or steroid use was defined as medication ended in 30 days before the index endoscopy.
